# Effectiveness of convolutional neural networks in the interpretation of pulmonary cytologic images in endobronchial ultrasound procedures

**DOI:** 10.1002/cam4.4383

**Published:** 2021-11-01

**Authors:** Ching‐Kai Lin, Jerry Chang, Ching‐Chun Huang, Yueh‐Feng Wen, Chao‐Chi Ho, Yun‐Chien Cheng

**Affiliations:** ^1^ Department of Mechanical Engineering College of Engineering National Yang Ming Chiao Tung University Hsin‐Chu Taiwan; ^2^ Department of Medicine National Taiwan University Cancer Center Taipei Taiwan; ^3^ Department of Internal Medicine National Taiwan University Hospital Taipei Taiwan; ^4^ Department of Internal Medicine National Taiwan University Hsin‐Chu Hospital Hsin‐Chu Taiwan; ^5^ Department of Computer Science College of Computer Science National Yang Ming Chiao Tung University Hsin‐Chu Taiwan

**Keywords:** benign and malignant classification, convolutional neural network, deep learning, endobronchial ultrasound, lung cytologic image, semantic segmentation

## Abstract

**Background:**

Rapid on‐site cytologic evaluation (ROSE) helps to improve the diagnostic accuracy in endobronchial ultrasound (EBUS) procedures. However, cytologists are seldom available to perform ROSE in many institutions. Recent studies have investigated the application of deep learning in cytologic image analysis. As such, the present study analyzed lung cytologic images obtained by EBUS procedures, and employed deep‐learning methods to distinguish between benign and malignant cells and to semantically segment malignant cells.

**Methods:**

Ninety‐seven patients who underwent 104 EBUS procedures were enrolled. Four hundred and ninety‐nine lung cytologic images obtained via ROSE, including 425 malignant and 74 benign, and most malignant were lung adenocarcinoma (64.3%). All the images were used to train a residual network model with 101 layers (ResNet101), with suitable hyperparameters selected to classify benign and malignant lung cytologic images. An HRNet model was also employed to mark the area of malignant cells. Automatic patch‐cropping was adopted to facilitate dataset preparation.

**Results:**

Malignant cells were successfully classified by ResNet101 with 98.8% classification accuracy, 98.8% sensitivity, and 98.8% specificity in patch‐based classification; 95.5% classification accuracy in image‐based classification; and 92.9% classification accuracy in patient‐based classification. Malignant cell area was successfully marked by HRNet with a mean intersection over union of 89.2%. The automatic cropping method enabled the system to complete diagnosis within 1 s.

**Conclusions:**

This is the first study to combine lung cytologic image deep‐learning classification with semantic segmentation. The model was optimized for high accuracy and the automatic cropping facilitates the clinical application of our model. The success in both lung cytologic images classification and semantic segmentation on our dataset shows a promising result for clinical application in the future.

## Graphical abstract

This study leveraged the high computational speed characteristics of deep learning to distinguish and segmentally mark malignant cells in lung cytologic images obtained by EBUS procedures. Classification with ResNet101 and segmentation with HRNet were completed within 1 s and with high accuracy.

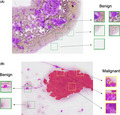

## 1 INTRODUCTION

Endobronchial ultrasound (EBUS) is a relatively new and minimally invasive procedure for diagnosing peripheral pulmonary lesions (PPLs) or mediastinal/hilar lesions.^1^, ^2^, ^3^ Prior research has confirmed its low procedure‐related complication rates.^4^ As a result, EBUS is widely applied in diagnosing thoracic lesions in many clinical institutions.^5^, ^6^ However, the diagnostic yields of EBUS alone are insufficient.^7^, ^8^ To improve the procedure's diagnostic accuracy, attempts have been made to combine EBUS with other methods, such as fluoroscopy, virtual bronchoscopic navigation, and electromagnetic navigation.^9^, ^10^, ^11^, ^12^, ^13^ However, the equipment employed in these procedures is not widely available because of the high cost and other limitations. As such, new approaches should be identified for clinical practice.

Rapid on‐site cytologic evaluation (ROSE) provides immediate feedback, which ensures correct and sufficient sample collection.^14^ Although ROSE can improve the diagnostic accuracy of EBUS procedures,^15^, ^16^, ^17^ it requires extra time from the cytologist and is largely considered economically inefficient.^14^ As such, few cytologists are willing to perform ROSE during a bronchoscopy procedure. Despite attempts to train pulmonologists to interpret cytologic smears on‐site while EBUS procedures are being performed,^8^ the EBUS procedure time appears to be prolonged because of the interruption of having to wait for the ROSE results. An adequate and effective means to present on‐site cytologic material during EBUS procedures is thus required if the procedure time is to be shortened.

With computer vision, machines can recognize and analyze images and videos, effectively allowing them to view the world as humans do. Advancements in computer vision with deep learning have led to considerable developments, particularly regarding convolutional neural networks (CNNs). Previous studies have applied CNNs in cytologic image analysis. Sanyal et al. identified papillary carcinoma on thyroid fine‐needle aspiration cytology smears^18^ and Savala et al. distinguished follicular adenoma from follicular carcinoma on fine‐needle aspiration of thyroid.^19^ Zejmo et al., Steiner et al., and Bejnordi et al. classified breast cancer cytologic specimen.^20^, ^21^, ^22^ Pouliakis et al. analyzed the role of artificial neural networks in cytopathology.^23^ Teramoto et al. classified lung cytologic images.^24^, ^25^ However, limited data have been reported on the application of CNNs in the presentation of lung cytologic specimens via EBUS procedures. Thus, the present study evaluated the accuracy of CNNs in distinguishing between malignant and benign pulmonary cytologic specimens obtained by EBUS procedures.

## 2 MATERIALS AND METHODS

### 2.1 Participants

Participants were 97 patients with 70 PPLs and 34 for mediastinal/hilar lesions who underwent EBUS procedures at the Division of Thoracic Medicine, National Taiwan University Cancer Center, or Division of Thoracic Medicine, National Taiwan University Hsin‐Chu Hospital, between November 2018 and February 2020. Participants comprised 53 men and 44 women aged 23–92 years (mean: 67.1 years) (Table 1). Written informed consent was obtained from each patient prior to the EBUS procedure. The study was approved by the National Taiwan University Cancer Center Institutional Review Board (IRB #202012053RINB).

**TABLE 1:** Characteristics of the patients and cytologic images

Characteristics	*n*
Patients	97
Age (years‐old, range)	67.1 (23–92)
Male (%)	53 (54.6)
Lesion location	104
Peripheral pulmonary lesions (%)	70 (67.3)
Mediastinal/hilar lesions (%)	34 (32.7)
Cytologic images	499
Malignancy (%)	425 (85.2)
Lung adenocarcinoma	321 (64.3)
Lung squamous cell carcinoma	41 (8.2)
Small cell lung cancer	33 (6.6)
Other NSCLC	12 (2.4)
Breast cancer	6 (1.2)
Pancreatic cancer	2 (0.4)
Hepatocellular carcinoma	10 (2.0)
Non‐malignant process (%)	74 (14.8)
Cryptococcosis	4 (0.8)
Granulomatous inflammation	2 (0.4)
Benign inflammation cells	55 (11.0)
Ciliated columnar cells	13 (2.6)

Abbreviation: NSCLC, non‐small cell lung cancer.

### 2.2 *EBUS procedures and on*‐*site cytologic image collection*

All EBUS procedures were performed by a pulmonologist, who has more than 10 years of experience in bronchoscopic clinical practice. Before the procedures, computer tomography images were screened for planning. For the diagnosis of PPLs, we performed EBUS‐guided transbronchial biopsy (EBUS‐TBB). If the target lesions were in the mediastinal or hilar area, EBUS‐guided transbronchial needle aspiration (EBUS‐TBNA) will be considered.

During EBUS‐TBB, we used flexible bronchoscopy (BF‐Q290 or BF‐1T290; Olympus Co.) combined with a 20‐MHz radial‐EBUS (UM‐S20‐17S or UM‐S20‐20R; Olympus Co.) for the procedure. The radial‐EBUS was inserted through the working channel of the scope into the suspected target bronchus based on computed tomography image. After confirming the location of the lesion, specimens were collected via biopsy forceps (NBF01‐11018120; MICRO‐TECH Co. Ltd.) or a guide sheath kit (K201/K203; Olympus Co.).

Convex‐EBUS (BF‐UC260FW; Olympus Co.) was dedicated for EBUS‐TBNA procedure. We identified the mediastinal and hilar lesions via slow withdrawal and rotation of the ultrasound transducer. TBNA biopsy with a 22‐gauge needle (NA‐201SX‐4022; Olympus Co.) was then performed to obtain histological cores.

During the procedure, material from the EBUS‐guided samples was imprinted on a clear glass slide without mounting coverslip for ROSE. Imprint smears were stained using a rapid method (Hemacolor; Merck KGaA) and evaluated on‐site via microscopy (BX43; Olympus Co.) by our pulmonologist who also has well cytologic training with more than 6‐year experience in cytologic clinical practice. When malignant cells were suspected during the ROSE, at least three more TBB, or one more TBNA would be performed at the same position. If none of the suspicious cells was detected, we would change to another site for repeating biopsy and ROSE study. The EBUS procedure would be terminated if no suspicious cell was explored via ROSE study for 2–3 times or if the patient could no longer tolerate the procedure. All tissue samples obtained by EBUS procedures were impregnated in 10% formalin, embedded in paraffin, and stained with hematoxylin and eosin for subsequent pathological analysis.

During the ROSE study, we also recorded the images of suspected malignant cells at 100×, 200×, or 400× amplification with a microscope digital camera system (DP22; Olympus Co.). Random cytologic images were also taken of samples with no malignant cells. Diagnosis of on‐site cytologic images was confirmed by the formal cytopathologic results. Based on the cytopathologic results, final diagnosis of malignancy was defined as positive and nonmalignant process was defined as negative in this study.

In the present study, 499 cytologic images were obtained from the participants, with 335 images via EBUS‐TBB and 164 images via EBUS‐TBNA. Four hundred and twenty‐five of them were classified as malignant and 74 as benign. In the 425 malignant images, 321 were lung adenocarcinoma, 41 were lung squamous cell carcinoma, 33 were small cell carcinoma, 12 were other non‐small cell lung cancer, 10 were hepatocellular carcinoma, 6 were breast cancer, and the remaining 2 were pancreatic cancer. Nonmalignant processes were pulmonary cryptococcosis in 4 cases, granulomatous inflammation in 2 cases, benign inflammation cells, which were dominant of alveolar macrophages, polymorphonuclear leukocytes, or lymphocytes in 55 cases, and ciliated columnar cells only in 13 cases (Table 1).

### 2.3 Data preprocessing

**FIGURE 1:**
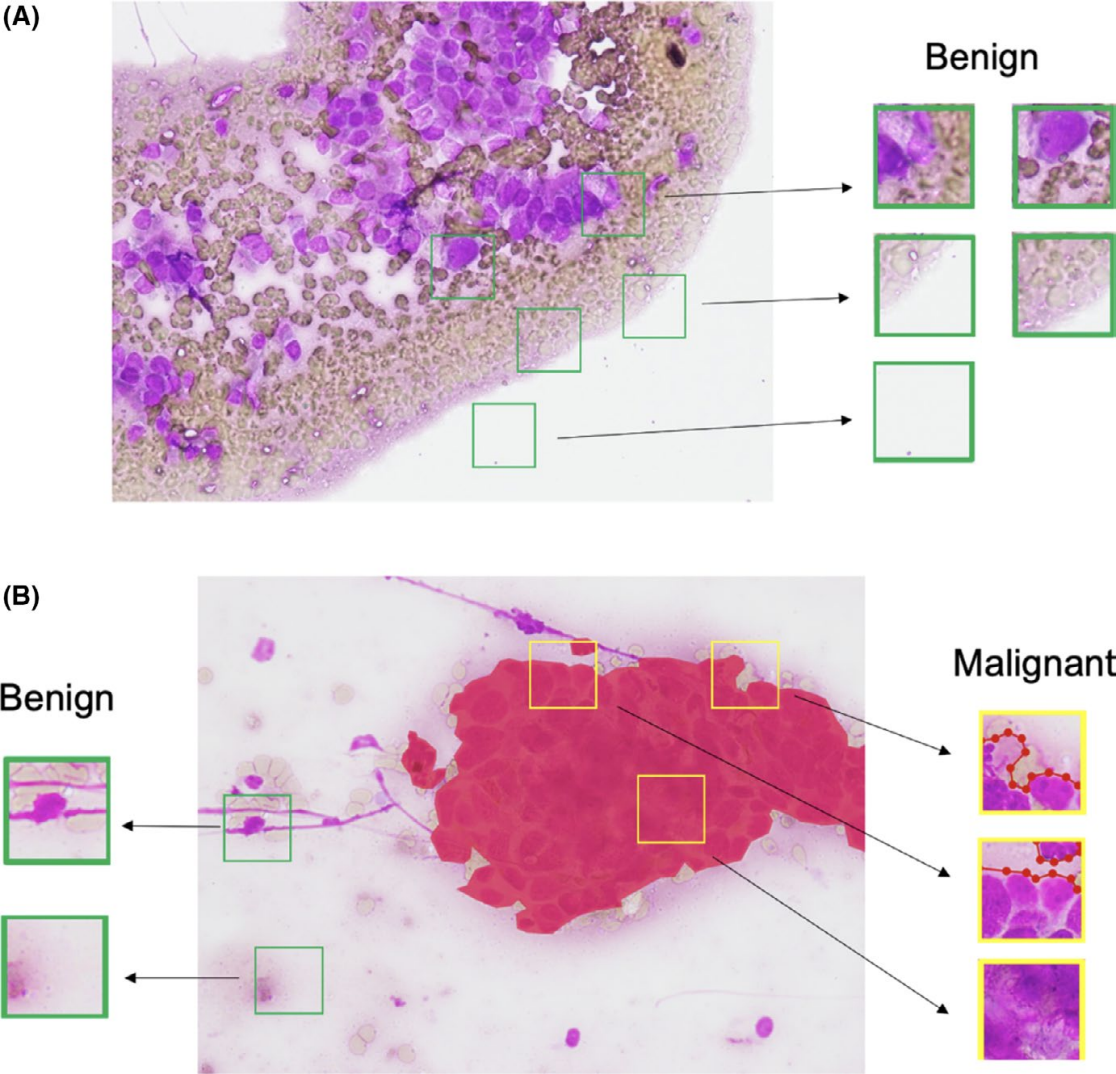
(A) Each benign image is randomly cropped into 15 benign patches 224 × 224 in size. (B) Each malignant image is randomly cropped into 10 malignant patches in areas overlapping malignant cells, and 5 benign patches in areas clear of malignant cells

The original images (1920 × 1440 pixels) were cropped into small patches (224 × 224 pixels). To balance the number of benign and malignant data, additional benign patches were generated from the benign images and nonmalignant cell areas in the malignant images. First, 15 benign patches were randomly cropped from the benign images (Figure 1A). Second, we labeled malignant cells area in malignant images with LabelMe^26^ in pixel level. From malignant images, 10 malignant patches were randomly cropped from areas overlapping malignant cells, and 5 benign patches were randomly cropped from areas clear of malignant cells (Figure 1B). A total of 7486 small patches were generated after automatic cropping, including 3286 benign patches and 4200 malignant patches. Finally, all the patches were divided into a training set (70% of participants), a validation set (15% of participants), and a test set (15% of participants).

### 2.4 Benign and malignant cell classification

The volume of training data was increased through data augmentation to avoid overfitting. Data augmentation included vertical flips, horizontal flips, random rotation by 20 degrees left and right, Gaussian blurring with a probability of 0.2 (SD: 0–3), contrast adjustment with a probability of 0.2 (gamma: 0.5–2), and hue and saturation adjustment with a probability of 0.2 (10 to −10 degrees).

Several deep‐learning classification models were constructed to evaluate their ability to classify benign and malignant patches. VGG16^27^ was built for comparison with research by Teramoto et al.^24^, ^25^ ResNet50, ResNet101, and ResNet152^28^ were selected due to its strong ability of avoiding accuracy saturation in deep networks. If the residual connection was the optimal path for backward propagation, then the network would approach the weight of the convolutional block to zero, making the network continue to learn with other residual blocks. ResNeXt50 and ResNeXt101^29^ were able to detect different scale size features by combining inception modules and residual connections. ResNeSt50, ResNeSt101, and ResNeSt200^30^ combined the ResNeXt model with an attention mechanism to enhance the ability of feature extraction. All the models were fine‐tuned for 100 epochs after pretraining on the ImageNet, dataset with natural color (RGB). The hyperparameters used in the benign and malignant patch classification were an initial learning rate of 0.0001, a batch size of 32, and image input size of 224. The optimizer was stochastic gradient descent, the loss function was binary cross‐entropy, and cosine learning rate decay was employed.

During testing, accuracy for patch‐based and image‐based classification was calculated separately. Image‐based classification accuracy was calculated using a sliding window algorithm, with the 224 × 224 patches sliding 112 pixels from the upper left corner of the image to the right, moving downward row‐by‐row. Since the benign patches contained background patches, which were easier to be classified, the model tended to overfit and focus less on classifying benign and malignant cells, resulting in a high false‐positive rate and low specificity. Moreover, classifying malignant patches was difficult and required distinguishing features such as the ratio of the nucleus to the cell, which can lead to a high false‐positive rate and low specificity. To solve these problems, we set the Softmax output threshold to 0.99. An image was classified as malignant only if there was at least a patch with a Softmax output higher than 0.99; otherwise, it was classified as benign.

For patient‐based classification, each patient had 2–10 images. A majority vote algorithm was thus employed to classify the cases as benign or malignant. The algorithm classified cases with more benign images than malignant images as benign, and vice versa for those with more malignant images. To avoid potentially missing malignant cases, those with the same number of benign and malignant images were also considered malignant.

### 2.5 Malignant cell segmentation

The CNN for semantic segmentation of malignant cells was trained with malignant images only. The 425 malignant images were divided into a training set (70% of images), a validation set (15% of images), and a test set (15% of images). Data augmentation was performed to increase the volume of data, including random rotation by 90 degrees, random horizontal flips, random vertical flips, hue saturation adjustment, brightness and contrast adjustment, and random cropping of images with a size of 1024 × 1024.

**FIGURE 2:**
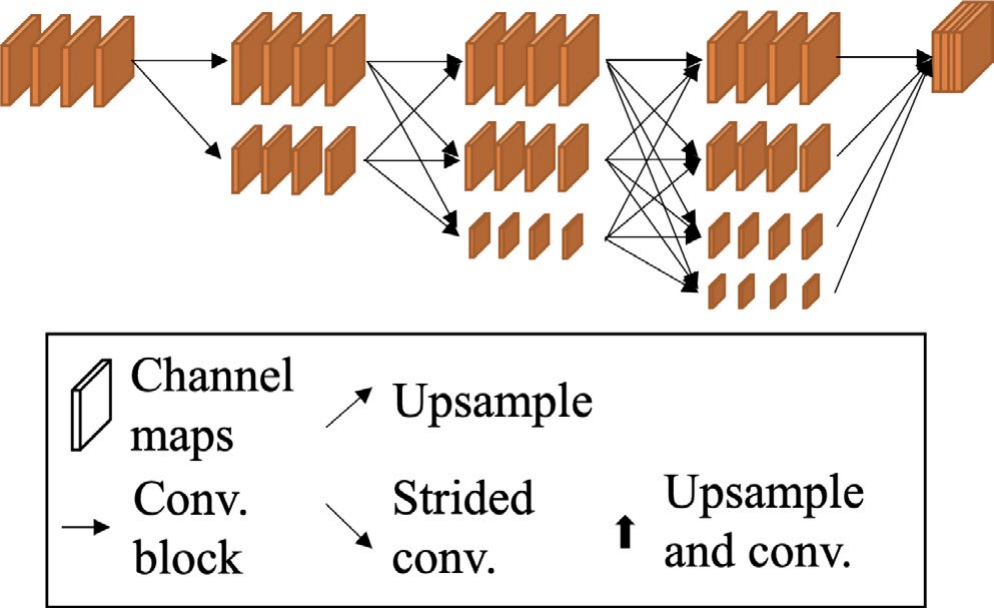
The diagram of HRNet. HRNet leveraged 256 × 256 high‐resolution image operations throughout the entire network and added some low‐resolution image information (128 × 128, 64 × 64, 32 × 32) at each stage to provide features of larger cells. This enabled the model to segment and distinguish malignant cells globally and locally

During training, different semantic segmentation models were constructed. FCN^31^ is the basic model with an encoder/decoder structure and was used as our baseline model. U‐Net^32^ is suitable for medical image segmentation which added skip connections between the encoder and decoder to achieve well performance with low parameters. PSPNet^33^ applies a pyramid pooling module after the encoder and is able to extract useful information in encoder. DeepLabv3^34^ is a powerful semantic segmentation model on semantic segmentation tasks which employed dilated convolution kernels to preserve high‐resolution information. DeepLabv3+^35^ simplified the decoder from DeepLabv3 to reduce the computational complexity while maintaining the ability of preserving high‐resolution information. FPN^36^ performed well on object detection tasks which stacked different sizes of feature maps in the decoder to obtain multiscale features. The design of the decoder of FPN can also perform well on semantic segmentation tasks. HRNet^37^ leveraged 256 × 256 high‐resolution image operations throughout the entire network and added some low‐resolution image information (128 × 128, 64 × 64, 32 × 32) at each stage to provide features of larger cells, as shown in Figure 2. This enabled the model to segment and distinguish malignant cells globally and locally. At the end of the network, feature maps of different sizes were stacked to obtain different levels of cell information. All the selected models were fine‐tuned for 300 epochs after pretraining on the ImageNet dataset. Hyperparameters employed for semantic segmentation included an initial learning rate of 0.001, batch size of 4, image input size of 1024, and weight decay of 0.0001. The optimizer was stochastic gradient descent, and the loss function was 0.5 times the binary cross‐entropy plus the dice loss.^38^ Cosine learning rate decay was employed.

### 2.6 Statistical analysis

Interpretations of the ResNet101‐based deep‐learning models were compared to the final diagnosis from the lung cytologic images. Sensitivity, specificity, positive predictive value, negative predictive value, and diagnostic accuracy rate were calculated according to standard definitions. Mean intersection over union (mIoU) was obtained for each semantic segmentation model during the malignant cell semantic segmentation process. The result for mIoU was calculated as the intersection area divided by the union area for a predicted area and target area. The formulas were as follows:

(1) $$\text{Sensitivity}=\text{True positives}/\left(\text{True positive}+\text{False negative}\right)$$

(2) $$\text{Specificity}=\text{True negatives}/\left(\text{True negative}+\text{False positive}\right)$$

(3) $$\text{Positive predictive value}=\text{True positives}/\left(\text{True positive}+\text{False positive}\right)$$

(4) $$\text{Negative predictive value}=\text{True negatives}/\left(\text{True negative}+\text{False negative}\right)$$

(5) mIoU=Area of intersection/Area of union

## 3 RESULTS

Among the models tested in this study, the ResNet101 model achieved excellent accuracy, sensitivity, and specificity in patch‐based classification, image‐based classification, and patient‐based classification of benign and malignant cells in lung cytologic images obtained via EBUS procedures. Our semantic segmentation tests also achieved a very high mIoU using the HRNet model.

A total of 66 images (990 patches) taken from 14 patients were used for testing. Table 2 shows that the sensitivity, specificity, positive predictive value, negative predictive value, and diagnostic accuracy rate of the ResNet101 model in patch‐based classification were 98.8%, 98.8%, 99.1%, 98.3%, and 98.8%, respectively. Table 3 shows that the patch‐based diagnostic accuracy rate of VGG16, ResNet50, ResNet152, ResNeXt101, ResNeSt50, ResNeSt101, and ResNeSt200 was 92.3%, 94.7%, 92.6%, 94.1%, 96.1%, 94.1%, and 91.7%, respectively. Image‐based classification using the patch‐based classification results of ResNet101 with sliding windows yielded 98.2% for sensitivity, 77.8% for specificity, 96.6% for the positive predictive value, 87.5% for the negative predictive value, and 95.5% for the diagnostic accuracy rate (Table 4). Tables S1 and S2 show the diagnostic accuracy rate of image‐based classification with cytologic images obtained via TBB and TBNA was 96.0% and 93.8%, respectively. For patient‐based classification using the image‐based classification results, we obtained 100% for sensitivity, 66.7% for specificity, 91.7% for the positive predictive value, 100% for the negative predictive value, and 92.96% for the diagnostic accuracy rate (Table 5). For semantic segmentation testing, the results for FCN, U‐Net, PSPNet, DeepLabv3, DeepLabv3+, and FPN were 81.3%, 84.2%, 78.6%, 88.2%, 87.0%, and 88.9% mIoU, respectively. The best result was 89.2% mIoU, obtained by the HRNet model (Table 6).

**TABLE 2:** Patch‐based benign and malignant classification results using ResNet101

ResNet101	Final cytologic image results		Total
Prediction	Positive	Negative	
Positive	563	5	568
Negative	7	415	422
Total	570	420	990

Note

Sensitivity =98.8%, specificity =98.8%, positive predictive value =99.1%, negative predictive value =98.3%, and diagnostic accuracy =98.8%.

**TABLE 3:** Patch‐based benign and malignant classification results using various deep‐learning classification models

Model	Accuracy (%)	Sensitivity (%)	Specificity (%)	Positive predictive value (%)	Negative predictive value (%)
VGG16	92.3	96.8	86.2	90.5	95.3
ResNet50	94.7	95.4	93.8	95.4	93.8
ResNet101	98.8	98.8	98.8	99.1	98.3
ResNet152	92.6	94.6	90.0	92.8	92.4
ResNeXt50	93.5	96.3	89.8	92.7	94.7
ResNeXt101	94.1	96.8	90.5	93.2	95.5
ResNeSt50	96.1	96.3	95.7	96.8	95.0
ResNeSt101	94.1	94.9	93.1	94.9	93.1
ResNeSt200	91.7	95.8	86.2	90.4	93.8

**TABLE 4:** Image‐based benign and malignant classification results based on the patch‐based classification results and a sliding window algorithm (EBUS‐TBB dataset + EBUS‐TBNA dataset)

ResNet101	Final cytologic image results		Total
Prediction	Positive	Negative	
Positive	56	2	58
Negative	1	7	8
Total	57	9	66

Note

Sensitivity =98.2%, specificity =77.8%, positive predictive value =96.6%, negative predictive value =87.5%, and diagnostic accuracy =95.5%.

**TABLE 5:** Patient‐based benign and malignant classification results based on the image‐based classification results and a majority vote algorithm

ResNet101	Final cytologic image results		Total
Prediction	Positive	Negative	
Positive	11	1	12
Negative	0	2	2
Total	11	3	14

Note

Sensitivity =100%, specificity =66.7%, positive predictive value =91.7%, negative predictive value =100%, and diagnostic accuracy =92.9%.

**TABLE 6:** Malignant lung cell semantic segmentation results using various deep‐learning models

Model	Backbone	mIoU (%)
FCN	ResNet101	81.3
U‐Net	ResNet101	84.2
PSPNet	ResNet101	78.6
DeepLabv3	ResNet101	88.2
DeepLabv3+	ResNet101	87.0
FPN	ResNet101	88.9
HRNet	HRNet	89.2
HRNet + OCR	HRNet	89.1

## 4 DISCUSSION

**FIGURE 3:**
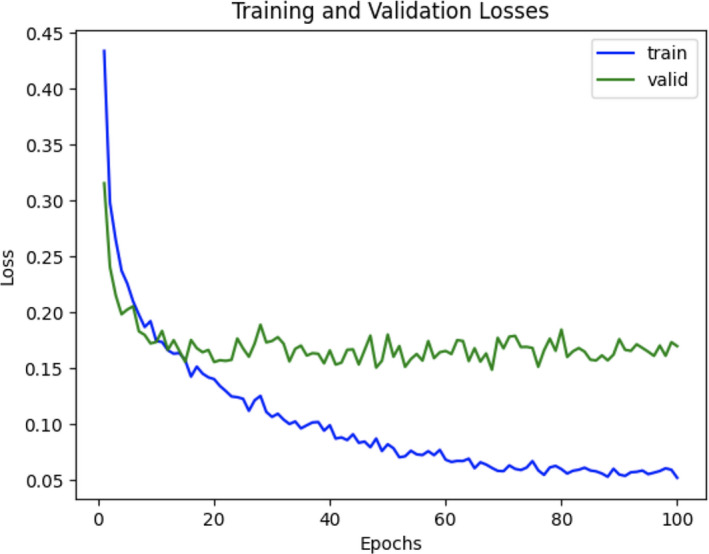
The loss/epoch curve of ResNet101 while fine‐tuning

A few studies have applied deep‐learning models in lung cytologic image classification and segmentation. In two studies, Teramoto et al have employed CNNs to classify benign and malignant cells from lung cytologic images, achieving 89.3% sensitivity and 83.3% specificity.^24^, ^25^ In our study, the ResNet101 model with patch‐based classification achieved 98.8% testing accuracy with 98.8% sensitivity and 98.8% specificity. The loss/epoch curve is shown in Figure 3. We also found that ResNet101 exhibited the highest accuracy, sensitivity, and specificity compared to the other CNN models (Table 3). By comparing the result of ResNet50, ResNet101, and ResNet152 in Table 3, we can see that residual connections in ResNet101 provide the network with appropriate model depth and size to learn distinguishable features from cells without overfitting. By comparing the result of ResNet and ResNeSt in Table 3, we can find that models with an attention mechanism have too many parameters, often leading to overfitting of the model. Thus, ResNet101 was optimal for learning most of the features for distinguishing between benign and malignant lung cytologic patches among the models we tested. Furthermore, we also observed that the patch‐based classification accuracy of ResNet101 can be increased from 92.2% to 98.8% using the data augmentation. The use of ImageNet dataset for transfer learning can also increase the patch‐based classification accuracy of ResNet101 from 86.5% to 98.8%.

**FIGURE 4:**
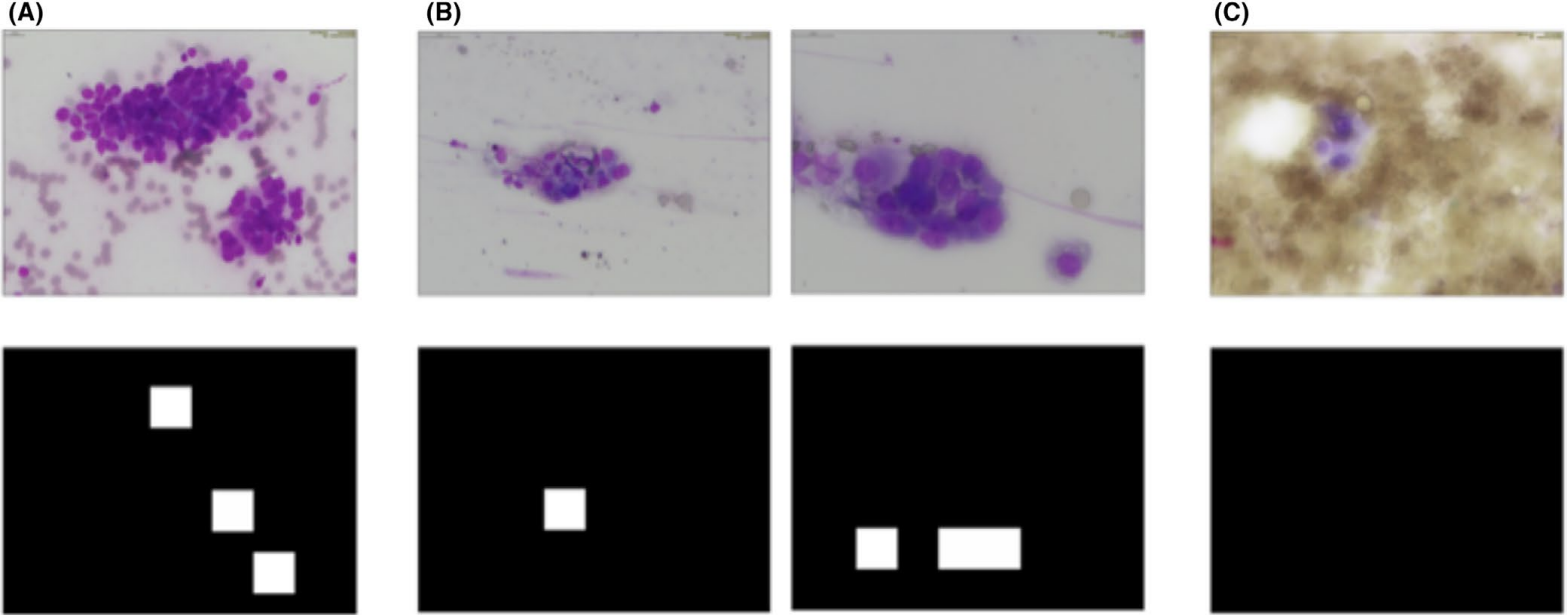
Image‐based classification results visualization. White areas are patches predicted as malignant and black areas are patches predicted as benign or background. (A) True‐positive image. (B) False‐positive images. (C) False‐negative images

In image‐based classification, images were used as a unit to distinguish between benign and malignant cases, with the images also cropped into patches through sliding windows for patch‐based classification. Since the patch‐based classification models tended to have low specificity and high false‐positive rate, as shown in Table 3, we set the threshold of Softmax output to be 0.99. If the Softmax output from the patch‐based classification was higher than 0.99, the image was classified as malignant. The image‐based classification accuracy was 95.5%, with 98.2% sensitivity and 77.8% specificity. Besides, the image‐based classification was also conducted on a dataset with cytologic images obtained via EBUS‐TBB only (Table S1) and a dataset with cytologic images obtained via EBUS‐TBNA only (Table S2). The diagnostic accuracy rate reached 96.0%, 93.8%, and 95.5% on the EBUS‐TBB dataset, EBUS‐TBNA dataset, and EBUS‐TBB dataset + EBUS‐EBNA dataset, respectively. To our knowledge, EBUS‐TBB and EBUS‐TBNA approach the different locations of the lesions, may have different cytologic pictures. The diagnostic accuracy of both study groups is very similar. The results demonstrated the effectiveness of our method on classifying cytologic images obtained via both EBUS‐TBB and EBUS‐TBNA. The classification results also showed that our model can perform well on both kinds of data obtained from these two different cytologic image acquisition methods. Table 4 shows there were two false positives and one false negative. The two false positives occurred because the nucleus was enlarged in these reactive benign cells (reactive bronchial cells and alveolar macrophages), which mimicked the appearance of malignant cells (Figure 4). The false‐negative image may have been caused by blurred cell boundaries, making it difficult for our CNN models to identify the target cells. The error in the image‐based classification of each patient accounted for only a few images, and the majority vote algorithm corrected these in the patient‐based classification.

The accuracy of the patient‐based classification was 92.9%, with 100% sensitivity and 66.7% specificity. The relatively low specificity may be due to the small sample size. Fourteen patients were enrolled for the test group, with one patient mistakenly categorized as a malignant case (Table 5). Although misdiagnosis might delay treatment planning in cancer patients, achieving 100% diagnostic accuracy in cytologic interpretation is difficult, even for experienced cytologists. Clinically, repeat sampling would be performed when lung malignancy is highly suspected based on computed tomography image finding or serological tumor marker elevation. We also found that among the two images from this patient, the cellular morphology in one image was very similar to that of malignant cells. We believe that obtaining more images to increase the number of training data during the EBUS procedure might minimize or eliminate this problem.

**FIGURE 5:**
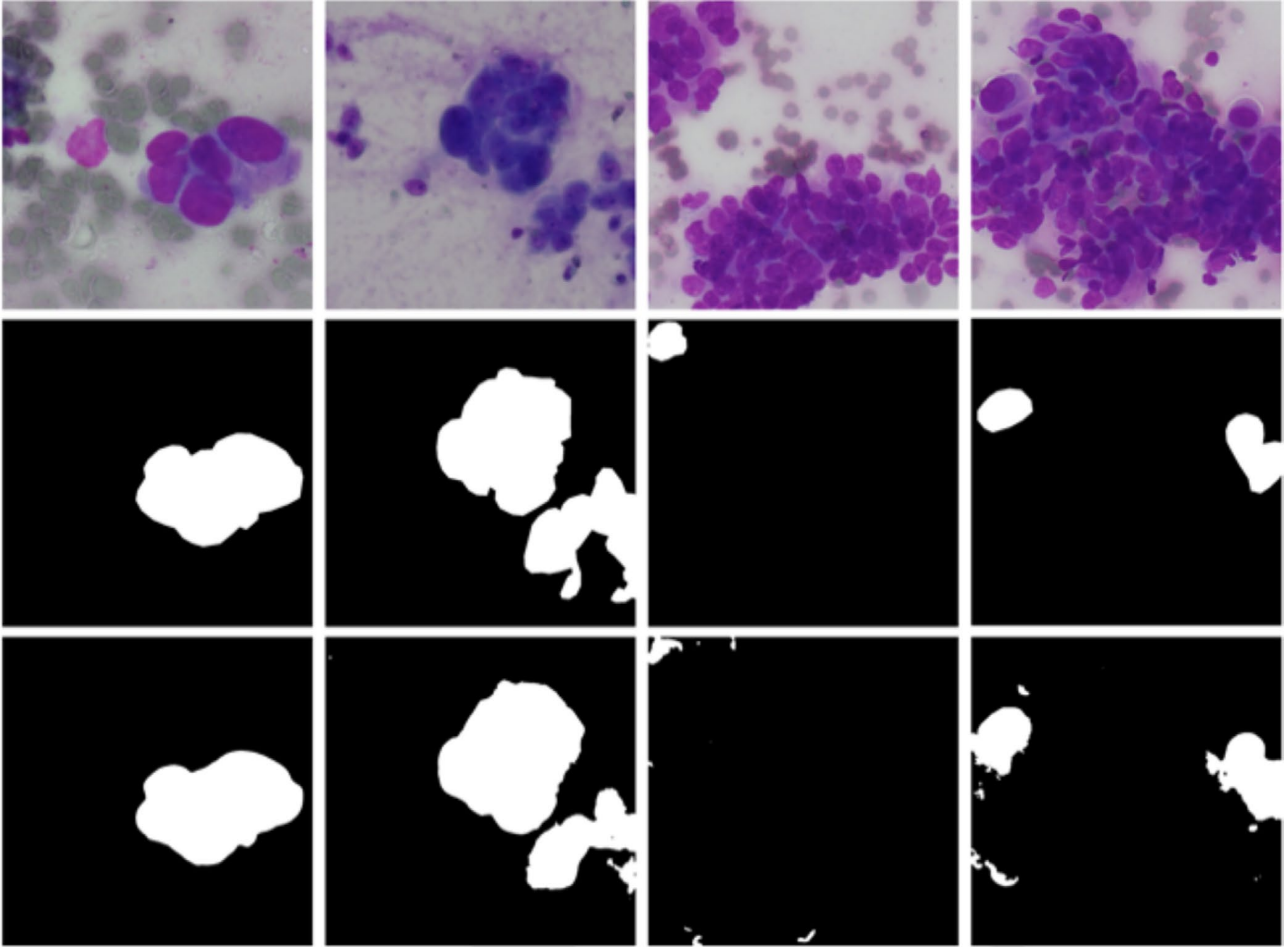
Semantic segmentation results visualization. Test images are in the first row; test targets (ground truth) are in the second row. White pixels denote areas predicted as malignant and black pixels denote areas predicted as benign or background. Semantic segmentation results are in the third row

The images classified as malignant by image‐based classification were sent to the semantic segmentation model to mark the malignant cell areas. This was performed using different models with adjusted hyperparameters. The best semantic segmentation result was achieved using HRNet (mIoU: 89.2%), as shown in Table 6. HRNet comprised four subnetworks. Each subnetwork was operated at different resolutions with information repeatedly exchanged with other subnetworks via multiscale fusion. We leveraged high‐resolution image operations throughout the entire network to focus on the features of small cells, and we added low‐resolution image information through multiscale fusion for the features of large cells. Hence, the model possessed sufficient information to segment and distinguish malignant cells, both globally and locally. Adding an object‐contextual representation (OCR) module^39^ to HRNet did not improve the accuracy, since the dataset in this study was a single‐class semantic segmentation task, which does not fully leverage the advantages of the OCR module. Figure 5 shows a comparison of the test images, test targets, and results of the semantic segmentation by HRNet (mIoU: 89.2%).

In our study, the image sample comprised considerably more malignant cases than benign cases. We leveraged a patch‐cropping method during data preprocessing to solve the data imbalance problem of image‐level data and then calculated the image‐based classification results based on the results of the patch‐based classification with a sliding window algorithm. The number of benign images was also directly upsampled to solve the data imbalance and was defined as “Image‐level + upsampling” in this study (Table 7). To compare the effect of different data preprocessing methods, we used ResNet101 with an initial learning rate of 0.0001, batch size of 32, optimizer set to stochastic gradient descent, and loss function set to binary cross‐entropy; cosine learning rate decay was also used. The only difference was that the input image size was 512 × 512 for the direct‐image classification and the patch size was 224 × 224 for the patch‐based classification. Experimental results showed that the accuracy of patch‐based classification with a sliding window was higher than the other two methods; thus, this approach could effectively solve any data imbalance. This overcomes the problem of directly classifying lung cytologic images with a data imbalance, which would result in the model predicting all the images as malignant. Even when the benign images were upsampled five times to balance the data, the benign data variation was still too low for the model to successfully learn the cell characteristics.

**TABLE 7:** Classification method comparison of different data preprocessing methods

Methods	Benign training data	Malignant training data	Accuracy (%)	Sensitivity (%)	Specificity (%)
Image‐level	59	324	86.4	100.0	0.0
Image‐level + upsampling	295	324	86.4	100.0	0.0
Patch‐based + sliding window	3286	4200	95.5	98.2	77.8

**FIGURE 6:**
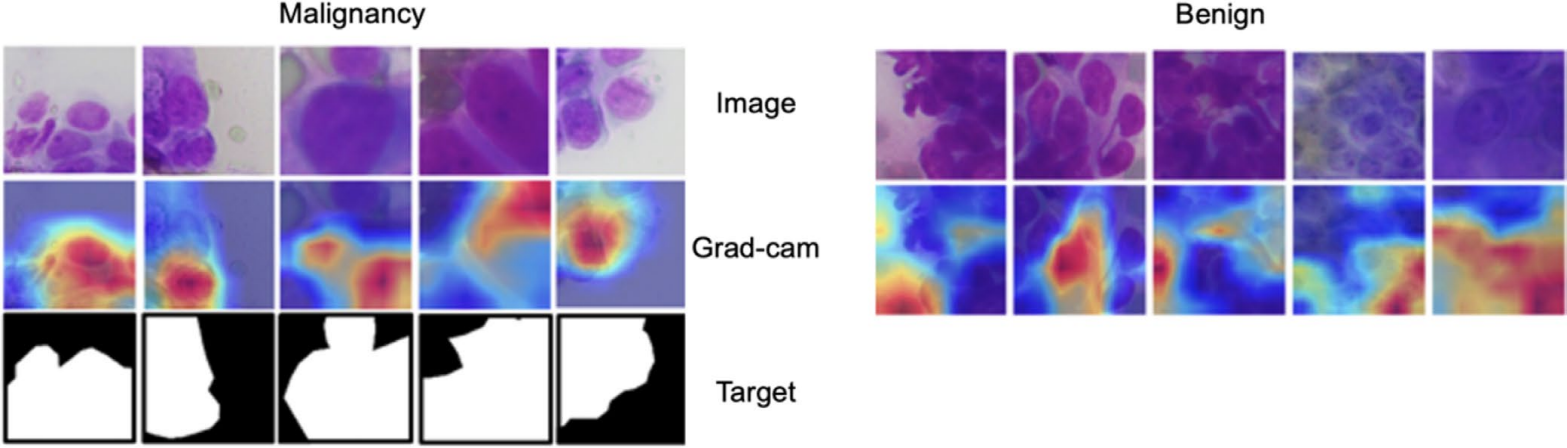
Patch‐based classification results visualization of true positives and true negatives using Grad‐CAM. Red areas show where the model focuses on learning; the blue area receives less focus

The weights of the last layer of the ResNet101 were also visualized for the patch‐based classification to confirm whether the model had focused on the correct information. The red area in Figure 6 indicates the area the model focuses on while learning; the blue area receives less focus. We found that the model learned the specific characteristics of malignant cells and ignored the background and benign areas, thus confirming that it focuses on the correct area of the cell.

We routinely perform ROSE with Hemacolor stain in our institution because the color is very similar to Diff‐Quik stain and the procedure time is shorter. Most reports on the efficacy of CNNs use Papanicolaou stain for cytologic preparation.^24^, ^25^ In previous clinical studies, different staining methods have been associated with sensitivities ranging from 72.8% to 96.9%.^40^, ^41^, ^42^ In the present study, ResNet101 exhibited excellent performance in differentiating between benign and malignant cells. This is the first study to use deep‐learning methods to interpret the cytologic specimens via Hemacolor stain, confirming that different staining methods can be used by deep‐learning models in interpreting cytologic specimens.

In future, more data and pulmonologist should join to overcome the limitations in our study. First, the volume of data was relatively small for training the deep‐learning model. Second, most of the malignant data were from cases of lung adenocarcinoma so the data amount of other cancer cells should be increased. Third, only one pulmonologist (L.C.K.) who is focus on interventional pulmonology has completed the course of cytologic training. Due to this reason, ROSE can only be performed during the bronchoscopy procedure and we limited our research to EBUS procedures. To overcome these limitations, future studies should follow the present investigation but with a larger and different study population.

In conclusion, classification procedures followed by semantic segmentation yield high accuracy for lung cytologic analysis. ResNet101 achieved 98.8% accuracy for patch‐based classification after hyperparameter adjustment. Image‐based classification accuracy was 95.5% with the sliding window algorithm, and patient‐based classification accuracy was 92.9%. After benign and malignant classification of lung cytologic images, semantic segmentation was employed to classify each pixel in the malignant images to mark malignant cell areas; for this, HRNet achieved an mIoU of 89.2%. This is the first study to combine lung cytologic image deep‐learning classification with semantic segmentation. It is also the first research and deep‐learning analysis of a dataset comprising Hemacolor‐stained lung cytologic images. We believe that the deep‐learning model employed in this study can be applied clinically in the interpretation of lung cytologic images in the future.

## CONFLICT OF INTEREST

The authors declare no conflict of interest.

## ETHICAL APPROVAL

The ethical approval was obtained from the institutional review board at National Taiwan University Hospital prior to commencing this study. REC number is 202012053RINB.

## Supporting information

Table S1‐S2

## Data Availability

The data that support the findings of this study are available upon request from the corresponding author. The data are not publicly available due to privacy or ethical restrictions.
