# Supplementary material for: Effectiveness of convolutional neural networks in the interpretation of pulmonary cytologic images in endobronchial ultrasound procedures
Source: Cancer Med. 2021 Nov 1;10(24):9047–57. doi: 10.1002/cam4.4383 (PMC8683546; doi:10.1002/cam4.4383)
Supplement: Supplementary file 1 — Table S1‐S2 [file CAM4-10-9047-s001.docx]

**SUPPLEMENTARY TABLE 1.** Image-Based Benign and Malignant Classification Results Based on the Patch-Based Classification Results and a Sliding Window Algorithm (EBUS-TBB dataset only)

| ResNet101 | Final cytologic image results | | Total |
| --- | --- | --- | --- |
| Prediction | Positive | Negative |  |
| Positive | 43 | 2 | 45 |
| Negative | 0 | 5 | 5 |
| Total | 43 | 7 | 50 |
| Sensitivity = 100%, specificity = 71.4%, positive predictive value = 95.6%, negative predictive value = 100%, diagnostic accuracy = 96.0%. | | | |

**SUPPLEMENTARY TABLE 2.** Image-Based Benign and Malignant Classification Results Based on the Patch-Based Classification Results and a Sliding Window Algorithm (EBUS-TBNA dataset only)

| ResNet101 | Final cytologic image results | | Total |
| --- | --- | --- | --- |
| Prediction | Positive | Negative |  |
| Positive | 13 | 0 | 13 |
| Negative | 1 | 2 | 3 |
| Total | 14 | 2 | 16 |
| Sensitivity = 92.9%, specificity = 100%, positive predictive value = 100%, negative predictive value = 66.7%, diagnostic accuracy = 93.8%. | | | |
